# Affective and Psychotic Disorders in War-Torn Eastern Part of the Democratic Republic of the Congo: A Cross-Sectional Study

**DOI:** 10.1155/2020/9190214

**Published:** 2020-07-24

**Authors:** Bives Mutume Vivalya, Germain Manzekele Bin Kitoko, Adelard Kalima Nzanzu, Martial Mumbere Vagheni, Rock Kasereka Masuka, Wilson Mugizi, Scholastic Ashaba

**Affiliations:** ^1^Department of Internal Medicine, Masereka General Hospital, North-Kivu, Democratic Republic of the Congo; ^2^Department of Psychiatry and Mental Health, Kampala International University Western Campus, Ishaka, Uganda; ^3^Department of Psychiatry, University of Kinshasa, Lemba, Kinshasa, Democratic Republic of the Congo; ^4^Department of Internal Medicine, Catholic University of Graben and Official University of Rwenzori, Butembo, Democratic Republic of the Congo; ^5^Faculty of Medicine, Catholic University of Graben, Butembo, Democratic Republic of the Congo; ^6^Postgraduate Studies and Research Directorate, Kampala International University Western Campus, Ishaka, Uganda; ^7^Department of Psychiatry, Mbarara University of Science and Technology, Uganda

## Abstract

**Background:**

There is lack of information about prevalence of affective and psychotic disorders triggered by traumatic events among people living in war-affected regions. This study is aimed at determining the prevalence rate of affective and psychotic disorders and the associated factors in a war-torn eastern part of Democratic Republic of the Congo.

**Methods:**

This epidemiological cross-sectional descriptive study was carried out from 1^st^ January 2019 to 31^st^ December 2019 at Cepima and Muyisa health centers. This study enrolled 344 patients that had experienced traumatic events in Eastern Democratic Republic of the Congo from the 1119 participants, of whom 229 had positive bipolar affective disorder and 115 patients had psychotic disorders.

**Results:**

The results revealed that bipolar affective disorders were two times more than psychotic disorders. Sexual abuse, sudden death of a relative, kidnapping, the physical torture, and childhood trauma were the psychological factors correlated to the occurrence of bipolar affective and psychotic disorders.

**Conclusions:**

It was concluded that the traumatic experiences were precursors for the occurrence of bipolar affective and psychotic spectrum disorders.

## 1. Background

Affective and psychotic disorders affect both men and women equally with a prevalence rate ranging between 1 and 3% in the general population [1]. These disorders can be triggered by traumatic events and are commonly reported among people in war- and conflict-affected areas [2]. Bipolar affective disorder is a mental disorder characterized by at least one or more manic or/and hypomania episodes with or without depression [3]. The prevalence rate of bipolar affective disorders found among populations in areas affected by war is usually higher than that in the nonaffected regions [4]. Priebe et al. [5] in a study examining the burden of mental disorders in war settings found out that prevalence of bipolar affective disorders with psychotic features ranged between 12.7 and 47.6% in the Balkans. Karam et al. [6] found a prevalence of bipolar affective disorders with psychotic features of 25.8% among a population of Lebanese affected by war. Bipolar affective disorder is among the five common mental disorders found among people living in areas affected by war with at least 20% in these areas presenting with mood symptoms, others being anxiety, posttraumatic stress disorder, and schizophrenia [7]. The common diagnoses in conflict-affected settings such as depression, posttraumatic stress disorder, anxiety disorders, and substance use disorders present features similar to those of bipolar affective disorders and psychotic disorders [8]. The long-term course of these disorders is complicated by potentially traumatic events [9, 10]. A comprehensive review of prevalence of bipolar disorders found the highest prevalence of bipolar affective disorder depressed among the studied population [11].

Further, psychotic features have been linked to depression and anxiety in 9% of the people with history of exposure to stressful life events. In conflict settings, studies have shown that both bipolar affective and psychotic disorders have social, psychological, or physical impairments and affect the quality of life and have been linked to premature death [1] in addition to low financial status, poor adherence to medication, and an elevated cost of living with a high risk of suicide [12]. Moreover, the psychological distress associated with armed conflict leads to stigma, low self-esteem, hopelessness, guilt, avoidance, and flashbacks which likely explain the psychotic symptoms commonly found among people with bipolar affective disorder [10]. The study by On'okoko et al. [13] which examined mental health in the Democratic Republic of the Congo (DRC) showed prevalence ranged between 6 and 31% for psychosis spectrum disorders bipolar disorder, compared to 13-23% for the affective disorders in conflict regions in the DRC. Given that mental health is commonly affected by psychological distress due to war, this study sought to determine the prevalence rates of affective and psychotic disorders and the associated factors among people with prior experience of armed conflict.

## 2. Methods

### 2.1. Participants

**Figure 1:**
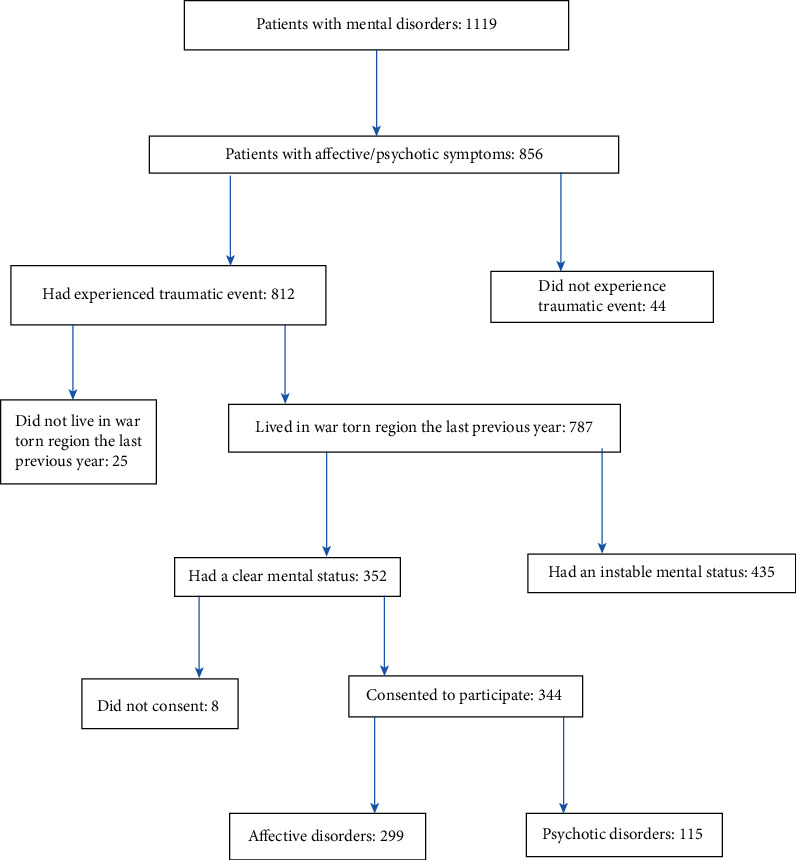
Participants' flow chart.

To carry out this epidemiological cross-sectional descriptive study, 1119 patients were screened between 1^st^ January 2019 and 31^st^ December 2019. Of these, 352 participants met the fifth edition of *Diagnostic and Statistical Manual of Mental Disorders* (DSM-5) criteria for bipolar affective and psychotic disorders [14]. Eight patients were excluded because they did consent to be involved in the study. In total, 344 participants were enrolled at two mental health centers in Butembo in DRC. All patients were admitted at Cepima and Muyisa mental health centers and that had a history of experiencing traumatic events aged above 18 years old, and who lived in the war-affected area for at least one year, and who had been clinically classified to have bipolar affective disorders or psychotic disorders [14]. Patients with affective or psychotic disorders who had not been exposed to armed conflict, those who had mood symptoms linked to trauma-related disorders such as posttraumatic stress disorder (PTSD), those aged less than 18 years old, and those who did not live in a war-torn area for the previous year before the study period were excluded (see Figure 1).

### 2.2. Ethical Approval

The study was approved by the Academic Board of the Catholic University of Graben. Permission to carry out the study was received from the Cepima and Muyisa mental health centers. A valid informed consent was received from the participants with clarity on their mental status and who are able to understand the content of the consent form. The study has been carried out according to the Helsinki Declaration.

### 2.3. Procedures

Two trained research assistants supervised by the first author collected data using a comprehensive questionnaire that consisted of the Mini International Neuropsychiatric Interview (MINI) version 6.0 and Harvard Trauma Questionnaire (HTQ) [15] as well as on sociodemographic characteristics and clinical factors such as sex, age, level of education attained, marital status, employment status, onset of mental disorders, had experienced a traumatic event, and having affective and/or psychotic symptoms. The questionnaire was translated from English to Kiswahili and then back translated into English to ensure clarity. This enabled the researchers to exclude participants who had mood symptoms of PTSD.

### 2.4. Data Analysis

The collected data were entered into STATA version 13 packages for statistical analysis. The prevalence of affective and psychotic disorders among participants was expressed as proportions of participants with the respective required diagnosis. Logistic regression was performed to assess the correlation between the factors associated with bipolar and psychotic disorders. The measure of association was odds ratios, and the threshold of statistical significance was set at 0.05.

## 3. Results

### 3.1. Sociodemographic and Clinical Factors Associated with Bipolar Affective Disorders and Psychotic Disorders

Table 1 shows sociodemographic and clinical factors of the participants extrapolated according their diagnosis (bipolar affective disorder and psychotic disorders). Most patients were males (64.6% versus 60.9%), with a sex ratio of 1.8 and 1.6, respectively. More than half (60.3%) of the participants with bipolar affective disorder were aged between 18 and 35 years old, compared to 50.4% of those with psychosis who were aged more than 35 years. Differences between the two groups were seen in the educational level attained, the marital status, employment status, and the onset of the mental disorders after a traumatic event. Of those with bipolar affective disorder, 30.1% had attained university school education compared to 44.9% of patients with psychotic disorders who had studied up primary school level (Table 1).

### 3.2. Sociodemographic Factors Correlated to Bipolar Affective Disorder versus Psychotic Disorder

Being female was significantly associated with both bipolar affective disorder (*p* value = 0.002) and psychotic disorder (*p* = 0.004). Also, having attained primary level of education was positively correlated to affective disorder (*p* value = 0.001) and psychotic disorder (*p* value = 0.003). Also short duration (6 months) following a traumatic was significantly associated with bipolar affective disorder (*p* value = 0.003) and psychotic disorder (*p* value ≤ 0.001 (Table 2).

### 3.3. Traumatic Events Experienced by the Participants

Majority (70.3%) of the participants with bipolar disorder had been exposed to a traumatic event, compared to 46% of patients with psychotic disorders. Traumatic events significantly associated with bipolar affective disorder included sexual abuse (OR: 0.6, 95% CI: 0.31-0.99, *p* value = 0.003), sudden death of a loved one (OR: 0.35, 95% CI: 0.12-0.84, *p* value = 0.040), and kidnapping (OR: 0.2, 95% CI: 0.04-1.43, *p* value = 0.035). On the other hand, traumatic events significantly associated with psychotic disorder included physical abuse (OR: 1.1, 95% CI: 0.39-2.01, *p* value = 0.012), sexual abuse (OR: 0.50, 95% CI: 0.22-1.19, *p* value ≤ 0.001), and childhood trauma (OR: 0.3, 95% CI: 0.21-0.85, *p* value = 0.022) (Table 3).

## 4. Discussion

The present study examined the relationship between the experienced traumatic events and onset of bipolar affective and psychotic disorders at two mental health centers in armed conflict-affected areas in DRC. The findings indicated that factors significantly associated with bipolar affective and psychotic disorders included sexual and physical abuses, childhood trauma, and kidnapping while the prevalence of bipolar affective disorder was higher than that of psychotic disorders among the participants.

This study evidenced that experiencing traumatic events in armed conflict-affected areas was followed by the occurrence of affective disorders two times more than the psychotic disorders. This finding is in accordance with the study of Palmieri et al. [16] who found a high prevalence of bipolar affective disorder (depressed phase) among people with history of experiencing a traumatic event. The possible explanation might be that the potentially traumatic effect is usually followed by the anxiety and depressive disorders which may fit the criteria of bipolar disorder with time.

Our results showed that the gender was associated with the affective disorders and psychotic disorders; especially, the female sex was more represented than the male. Similarly, Belteczki et al. [17] illustrated that 66% of participants were the female in German. Despite the variation across the study methodology and the participant's sociodemographic and clinical factors, the gender is often observed.

We observed that the majority of participants diagnosed with bipolar affective disorder were married, practiced business and farmer jobs, and had attained secondary and university levels compared to those with psychotic disorders. This is in agreement with a number of studies which investigated the rates of bipolar affective disorder according to the sociodemographic variables [18]. There is evidence that high level of education, the married status, and the employment were associated with increase of bipolar disorder than schizophrenia [19]. Furthermore, our findings are similar to previous research showing high level of education attained and high employment rates among patient with bipolar affective disorder compared to those with psychotic disorders [14].

Additionally, our findings showed a strong association between the female sex and the early onset and the bipolar affective and psychotic disorders. This is in agreement with results of Charlson et al. [2] who found an early onset among mental patients, especially female participants with history of traumatic event. This difference could be explained by the presentation and clinical course of bipolar depending on age of onset, associated with the high rates of bipolar affective disorder found by this study.

There is also emerging evidence that bipolar affective disorder in the depressive phase was the common bipolar affective disorder among the study participants, while schizoaffective disorder was the most common psychotic disorder detected. These results are in contrast to most previous studies which found a higher prevalence of mania and hypomania [1, 20]. The prolonged traumatic experiences may explain the different prevalence rates of psychotic disorders and bipolar affective disorders among participants.

In contrast to other studies, traumatic events were found more among patients with bipolar affective disorder compared to those with psychotic disorders [21]. Our study demonstrates that majority of the participants with bipolar affective disorder have been exposed to a stressful life event (70.3%), comparing to 46% of those with psychotic disorders. While the evidence is stronger for schizophrenia after a potentially traumatic disorder, few studies found the link between trauma and bipolar affective disorder in long term. The explanation is that trauma-related disorders are considered than bipolar affective disorders.

Our findings revealed that the physical abuse was significantly associated with bipolar affective disorder (OR: 1.1, 95% CI: 0.39-2.01, *p* value = 0.012) comparing to the sexual abuse, kidnapping, and sudden death of a loved person which were more correlated to the occurrence of psychotic disorders. These results are in contrast with other studies which found a strong correlation between sexual abuse and occurrence of bipolar disorder. The cumulative trauma was more correlated to psychotic disorder than affective disorders by several researchers [22, 23], which focused on the predictors on mental disorders. Also, the statistically significant association between physical abuse and bipolar affective disorders could be explained by the maltreatment of civilians in war settings and the associated stress which leads to psychological distress [24]. The strong correlation between childhood trauma and psychotic disorders found in our study is similar to prior researches [17, 25] which suggested that the prolonged exposure to trauma is linked to depression with psychotic-like symptoms. Moreover, the loss of a relative or a loss a job has been reported to the trigger a transient mood state which is the trigger of affective disorders [26]. The psychopathology induced by change is neurobiology which is commonly suggested by the consequences of trauma in early life.

### 4.1. Study Strengths

This study is the first documented study carried out in the war-torn area eastern of the Democratic Republic of the Congo. Its findings are an additional update to the knowledge on major psychiatric disorders following the traumatic events in an armed conflict setting.

### 4.2. Study Limitations

We could not establish causal-effect relationship given the cross-sectional descriptive study. Also, given that the onset of mental illness is within 6 months, the similarity between mood disorders and PTSD leads to exclude the trauma-related disorders based on clinical features of trauma-related disorders.

## 5. Conclusions

Traumatic events were significantly associated with affective disorder than psychotic disorders. Bipolar affective disorder depressed phase and the schizoaffective disorder were the common disorders found among the study participants. The findings suggest that physical torture, sexual abuse, childhood trauma, sudden death of a loved one, and kidnapping may be risk factors for occurrence of affective and psychotic disorders among people who have experienced war and conflict. The study findings emphasize the impact of exposure to conflict and onset of the bipolar affective and psychotic disorders.

## Figures and Tables

**Table 1:** Sociodemographic and clinical characteristics.

Variables		Bipolar affective disorders		Psychotic disorders	
		Frequency	Percent	Frequency	Percent
Gender	Sex ratio	1.8		1.6	
	Male	148	64.6	70	60.9
	Female	81	35.4	45	39.1

Age	Mean	26.7 SD: 8.7		27.4 SD: 7.1	
	18-35 years	138	60.3	57	49.6
	More than 35 years	91	39.7	58	50.4

Educational level attained	Illiterate	4	1.8	16	13.3
	Primary	77	33.6	51	44.9
	Secondary	79	34.5	30	26.1
	University	69	30.1	18	15.7

Marital status	Single	79	34.5	59	51.3
	Married	90	39.3	14	12.2
	Widowed	29	12.7	10	8.7
	Separated	31	13.5	32	27.8

Employment status	Unemployed/retired	47	20.5	54	50
	Health worker	42	18.3	6	5.2
	Student	49	21.4	13	11.3
	Others	91	39.7	42	36.5

Onset of the illness	6 months	16	7	21	18.3
	7 months–5 years	172	77.3	71	61.7
	Up to 5years	41	17.9	23	20

**Table 2:** Correlated factors between affective disorder and psychotic disorder.

		Bipolar affective disorders		Psychotic disorders	
Gender	Male	148		70	
	Female	81	0.002	45	0.004

Age	18-35 years	138		57	
	Up to 35 years	91	0.974	58	0.238

Educational level attained	Illiterate	4		16	
	Primary	77	0.001	51	0.003
	Secondary	79		30	
	University	69		18	

Marital status	Single	79		59	
	Married	90	0.175	14	0.097
	Widowed	29		10	
	Separated	31		32	

Employment status	Unemployed/retired	47	0.800	54	0.946
	Health worker	42		6	
	Student	49		13	
	Others	91		42	

Onset of the illness	6 months	16	0.003	21	<0.001
	7 months–5 years	172		71	
	Up to 5years	41		23	

**Table 3:** Potential traumatic events experienced by the participants.

Traumatic event	Bipolar affective disorders				Psychotic disorders			
	Yes (%)	No (%)	OR 95% CI	*p* value	Yes (%)	No (%)	OR 95% CI	*p* value
Sexual abuse	24 (15)	9 (13.2)	0.6 (0.31-0.99)	0.003	5 (8.9)	13 (22)	0.5 (0.22-1.19)	<0.001
Accident	38 (23.6)	15 (22.1)	1.0		9 (16.1)	12 (20.3)	1.0	
Sudden death of relatives	11 (6.8)	14 (20.6)	0.35 (0.12-0.84)	0.040	3 (5.4)	8 (13.6)	0.6 (0.34-0.99)	0.201
Loss of job	29 (18)	4 (5.9)	1.2 (0.47-3.07)	0.098	4 (7.1)	9 (15.3)	1.3 (0.51-3.18)	
Kidnapping	23 (14.3)	12 (17.6)	0.2 (0.04-1.43)	0.035	10 (17.9)	7 (11.9)	0.7 (0.28-2.01	0.706
Imprisonment	11 (6.8)	5 (7.4)	1.0 (0.41-2.33	0.234	2 (3.6)	2 (3.4)	1.0	0.065
Physical abuse	14 (8.7)	6 (8.8)	1.1 (0.39-2.01)	0.012	9 (16.1)	3 (5.8	0.7 (0.26-1.94)	0.026
Incurable illness	2 (1.2)	1 (1.5)	1.0		2 (3.6)	2 (3.4)	0.3 (0.14-0.72	0.607
Childhood trauma	9 (5.6)	2 (2.9)	0.7 (0.21-0.89)	0.651	12 (21.4)	3 (5.1)	0.3 (0.21-0.85)	0.022
Total	161 (70.3%)	68			56 (46.7)	59		

The factors that were significantly associated with psychotic disorders were childhood trauma (OR: 0.3, 95% CI: 0.2-0.85, *p* value = 0.022), physical abuse (OR: 0.7, 95% CI: 0.26-1.94, *p* value = 0.02), and sexual abuse (OR: 0.5, 95% CI: 0.22-1.19, *p* value ≤ 0.001).

## Data Availability

The data used to support the findings of this study are available from the corresponding author upon request.

## Abbreviations

DRC: Democratic Republic of the Congo
DSM: Diagnostic and Statistical Manual of Mental Disorders
HTQ: Harvard Trauma Questionnaire
MINI: Mini International Neuropsychiatric Interview
PTSD: Posttraumatic stress disorder
OR: Odd ratio
STATA: Statistical software package health care, medical.
